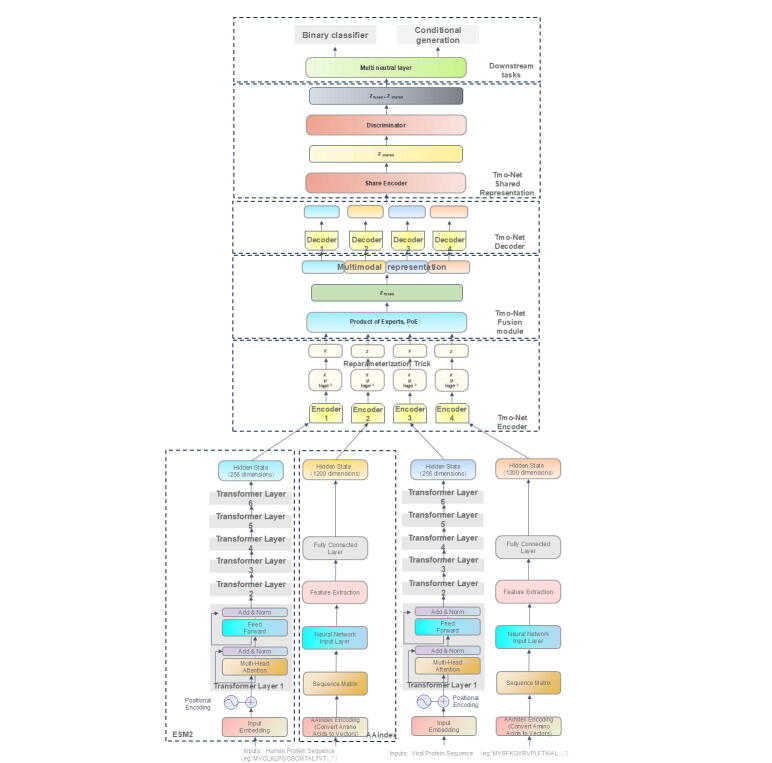# DeepHVI: A multimodal deep learning framework for predicting human-virus protein-protein interactions using protein language models

**DOI:** 10.1016/j.bsheal.2025.07.005

**Published:** 2025-07-11

**Authors:** Xindi Wang, Junyu Luo, Xiyang Cai, Ruibin Liu, Yixue Li, Chitin Hon

**Affiliations:** a Faculty of Innovation Engineering, Macau University of Science and Technology, 999078, Macao Special Administrative Region of China; b Institute of Systems Engineering, Macau University of Science and Technology, 999078, Macao Special Administrative Region of China; c Guangzhou National Laboratory, Guangzhou International Bio Island, Guangzhou 510005, China; d GZMU-GIBH Joint School of Life Sciences, The Guangdong-Hong Kong-Macau Joint Laboratory for Cell Fate Regulation and Diseases, Guangzhou Medical University, Guangzhou 511436, China; e Key Laboratory of Systems Health Science of Zhejiang Province, School of Life Science, Hangzhou Institute for Advanced Study, University of Chinese Academy of Sciences, Hangzhou 310024, China; f School of Life Sciences and Biotechnology, Shanghai Jiao Tong University, Shanghai 200240, China; g Shanghai Institute of Nutrition and Health, Chinese Academy of Sciences, Shanghai 200030, China

**Keywords:** Protein-protein interaction, Virus, Protein language model, Multimodal fusion

## Abstract

Understanding human-virus protein-protein interactions is critical for studying molecular mechanisms driving viral infection, immune evasion, and propagation, thereby informing strategies for public health. Here, we introduce a novel multimodal deep learning framework that integrates high-confidence experimental datasets to systematically predict putative interactions between human and viral proteins. Our approach incorporates two complementary tasks: binary classification for interaction prediction and conditional sequence generation to identify interacting protein partners. By leveraging protein language models and multimodal fusion, the framework demonstrates improved accuracy in identifying biologically relevant interactions. For empirical validation, we applied this method to predict severe acute respiratory syndrome coronavirus 2 (SARS-CoV-2)-human interactions, identifying candidate proteins absent from training data, several of which were corroborated by independent studies. These predictions offer critical insights into potential therapeutic targets, facilitating the design of antiviral drugs and vaccines. By enabling rapid, cost-effective discovery pipelines, our study contributes to pandemic preparedness and public health interventions, underscoring its value in combating emerging infectious diseases.

## Highlights

**Scientific questions:** Deciphering human-virus protein-protein interactions (HV-PPIs) is essential for understanding viral infections and development therapeutic interventions. Predicting HV-PPIs remains a formidable challenge due to the complexity of viral proteins, many of which are poorly characterized. This study proposes a multimodal deep learning framework capable of predicting HV-PPIs and identifying potential interacting partners for uncharacterized viral proteins. By leveraging protein sequence embeddings alongside complementary features derived from both human and viral proteins, the framework improves prediction accuracy, providing a powerful tool to advance research in virology and host-pathogen interactions.
**Evidence before this study:** Current methods predominantly rely on sequence-based or structure-based models, often neglecting multimodal protein features. Few studies have integrated both binary classification and sequence generation in the prediction of protein interactions.
**New findings:** The DeepHVI framework improves HV-PPI prediction by integrating deep learning with multimodal strategies. Utilizing protein language models and physicochemical properties, it captures complex biological signatures, enhancing prediction reliability. A multimodal fusion architecture enables robust interaction predictions. The framework features two tasks: binary classification for host-viral protein interactions and conditional sequence generation to predict potential interactors. This dual-task design overcomes traditional PPI limitations and enhances efficiency, making it adaptable for emerging epidemics.
**Significance of the study:** Viral infections represent a major public health threat, and the incomplete characterization of viral proteins hinders our understanding of pathogenesis and the development of effective therapies. HV-PPIs are essential for viral replication and immune evasion, yet their experimental characterization remains challenging. This study introduces DeepHVI, a multimodal deep learning framework that integrates protein sequence data to predict HV-PPIs, thereby advancing viral research and enhancing public health preparedness.

## 1 Introduction

Viral infections represent a persistent threat to global public health, underscoring the urgency of biomedical research in this field. While virology has advanced significantly in recent decades, the functional characterization of viral proteins remains incomplete [1,2]. This knowledge gap hampers the understanding of viral pathogenesis, particularly in how viral proteins contribute to the mechanisms of disease. A crucial aspect of viral pathogenicity involves human-virus protein-protein interactions (HV-PPIs), where viral proteins co-opt host cellular machinery to facilitate replication, evade immune surveillance, and promote transmission [1,3,4]. Therefore, a deeper characterization of these viral proteins is essential to understanding their role in pathogenicity and developing targeted therapeutic interventions and prophylactic vaccines. Consequently, systematic investigation of HV-PPIs provides a strategic framework for understanding viral protein functions. Such analyses enable functional inference of uncharacterized viral proteins, identification of host pathways vulnerable to viral exploitation, and mechanistic understanding of cellular subversion strategies. These insights could inform novel antiviral therapies by targeting critical host-pathogen interaction nodes.

However, the experimental characterization of HV-PPIs in emerging pathogens presents significant challenges. Methodological constraints, such as time-consuming optimization processes, validation bottlenecks, and insufficient baseline datasets, hinder rapid response capabilities before and during outbreaks [[5], [6], [7]]. To address these limitations, computational HV-PPI prediction approaches have been developed [[8], [9], [10], [11]]. However, most applications have proven less effective compared to the human-human protein-protein interaction (PPI) prediction, primarily due to the complexity of virus proteins and the inherent limitations of existing methodologies. Viral proteins display structural dynamism and conformational diversity when interacting with host cellular machinery [12], features that conventional computational models which typically rely on homology-based algorithms and statistical methods (e.g., template-based docking, sequence alignment methods, or shallow machine learning frameworks) struggle to accurately capture the intricate patterns and non-linear relationships inherent in complex biological systems. Deep learning methods have emerged recently and can uncover hidden patterns that conventional models may miss, but they heavily rely on existing data from comprehensively annotated protein interaction databases; however, a substantial fraction of viral PPIs remains uncharacterized or incompletely mapped [13], introducing significant gaps in existing knowledge bases and undermining predictive accuracy. Consequently, the persistent gap between pathogen emergence and effective interventions critically hinders public health preparedness, underscoring the urgent need for advanced computational frameworks capable of robust, generalizable predictions in HV-PPIs [14,15].

Protein language models (pLMs) have recently emerged as transformative tools in biological research [16]. Pre-trained on large protein sequence datasets, pLMs capture biological and evolutionary insights from protein sequences. Unlike traditional approaches that require explicit structural templates or manually engineered features, intelligent models (e.g., deep learning-based pLMs) learn latent representations directly from raw sequence data [[17], [18], [19]]. This capability enables them to capture evolutionary constraints and functional signatures without relying on prior structural annotations, thereby addressing the conformational plasticity of viral proteins. [20,21]. By incorporating experimental data and biological knowledge, these models facilitate high-throughput predictions of protein functions and interactions. Building on these developments, recent studies have tried to incorporate multimodal data beyond primary protein sequences, such as three-dimensional structural features and physicochemical properties, into pLM frameworks. This multimodal integration enables models to capitalize on the complementary relationships between data types, thereby mitigating the constraints associated with individual data modalities. For instance, combining sequence data with structural insights improves predictions of protein functions [22,23]. Such advancements underscore the potential of pLMs to serve as versatile platforms for integrative biological discovery, bridging gaps between sequence-based predictions and experimentally validated functional insights.

In this study, we present DeepHVI, a multimodal deep learning framework designed to predict HV-PPIs from amino acid sequences. The model uses hierarchical feature extraction from human and viral protein sequences, integrated with multimodal learning, to identify specific and distinguishable patterns in interacting virus-host protein pairs. These patterns represent unique sequence-based or interaction-based characteristics that differentiate interacting protein pairs from non-interacting ones. By learning these patterns, DeepHVI can predict potential interactions for unconfirmed protein pairs. Furthermore, the framework addresses scenarios where viral protein functions are entirely uncharacterized by generating candidate interacting protein sequences. This approach enables researchers to infer potential roles of viral proteins based on their predicted interaction partners, thus providing a computational tool for advancing research on viral pathogenesis and host-pathogen dynamics.

## 2 Materials and methods

### 2.1 Data acquisition and curation

PPIs between viruses and hosts are primarily governed by biophysical and biochemical properties derived from their amino acid sequences. Key factors include: (1) structural complementarity: the three-dimensional shape of protein surfaces determines whether two proteins can physically bind; (2) complementary regions (e.g., pockets and protrusions) enable stable docking; (3) electrostatic interactions: oppositely charged residues (e.g., positively charged arginine/lysine and negatively charged aspartate/glutamate) form salt bridges that stabilize binding; (4) hydrophobic patches: clusters of nonpolar residues (e.g., leucine, valine) drive interactions by minimizing exposure to aqueous environments; (5) hydrogen bonding: polar groups (e.g., –OH, –NH) form directional bonds that enhance binding specificity.

To capture these factors, we constructed a dataset from two publicly available databases: Human–Virus Interaction Data Base (HVIDB) [21] and Universal Protein (UniProt) [22]. We retrieved protein sequences by matching the protein ID names from HVIDB with the protein names in the UniProt database. In cases where naming inconsistencies occurred and the corresponding protein sequences could not be identified, we excluded such data from further analysis. Ultimately, 45,427 experimentally validated HV-PPI pairs were retained, involving 6,809 human proteins and 1,972 viral proteins from 556 viral species (covering both deoxyribonucleic acid [DNA] / ribonucleic acid [RNA] viruses). Negative samples (22,713 pairs) were generated by randomly pairing human and viral proteins with no documented interactions, maintaining a 1:0.5 positive-to-negative ratio. This balance allows the model to learn subtle discriminative features (e.g., charge distribution, hydrophobic motifs) critical for distinguishing true interactions from non-interacting pairs. The dataset was split into training (80 %) and test (20 %) sets to evaluate performance.

### 2.2 Whole model architectures

**Fig. 1:**
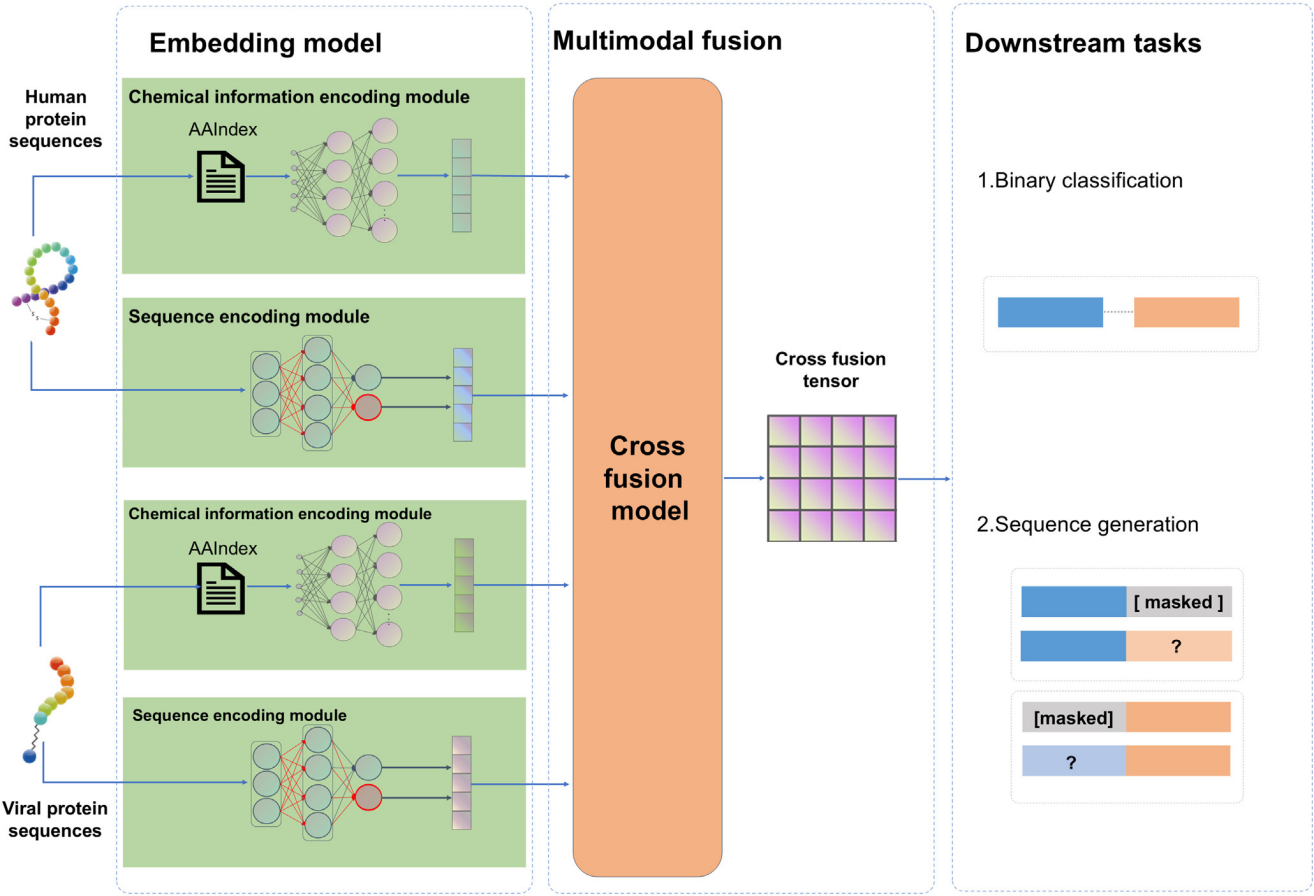
Architectural overview of DeepHVI. The framework comprises three primary components: (1) the embedding module, which encodes raw sequence input data into feature representations; (2) the multimodal fusion module, responsible for integrating heterogeneous data modalities; and (3) the downstream task module, which executes task-specific objectives such as prediction or classification.

The DeepHVI framework consists of three core modules (Fig. 1): the embedding module, which extracts protein features by capturing both sequence and structural characteristics using representation learning techniques; the multimodal fusion module, which integrates multimodal features to enhance overall performance; and the downstream task module, which addresses specific bioinformatics applications, enabling effective use in real-world scenarios. For the downstream task module, we designed two tasks: (1) a binary classification task predicting interaction between a pair of human and viral proteins, and (2) a conditional sequence generation task generating interacting protein partners for a given input sequence. For the classification task, the model takes as input the representations of both human and viral protein sequences and predicts whether the pair interacts, thereby facilitating the identification of potential host-pathogen protein-protein interactions. For the sequence generation task, given a human or viral protein sequence as input, the model outputs candidate interacting partners drawn exclusively from naturally occurring proteins. The detailed architecture of the model is shown in Fig.S1. To address biosafety and ethical considerations, the framework restricts sequence generation to natural proteins, preventing the generation of artificial protein sequences not found in nature.

### 2.3 Embedding module

The embedding module encodes amino acid sequences and biochemical properties into vector representations, capturing structural, functional, and evolutionary characteristics of proteins. For sequence feature extraction, we employed two distinct pLM frameworks: ESM-2 [23] and LucaProt [24]. Additionally, physicochemical properties were derived using AAindex [25].

ESM-2 processes protein sequences analogously to natural language. Each amino acid is mapped to an embedded vector, and a self-attention mechanism enables the model to analyze pairwise residue interactions, capturing both local motifs and long-range dependencies. The resulting high-dimensional vectors encapsulate global structural and functional information [23]. We chose the esm2_t6_8M_UR50D version to generate feature representations of protein sequences. This architecture processes amino acid sequences with a maximum length of 1,024 residues. For sequences shorter than this limit, we applied padding to achieve uniform input dimensions; for sequences exceeding the limit, truncation was performed to retain the initial 1,024 amino acids. The encoder produces 320-dimensional embeddings, while the decoder accepts 256-dimensional inputs, enabling efficient compression of sequence information into low-dimensional, context-aware embeddings. These embeddings capture structural and functional attributes of both human and viral proteins for downstream analysis.

LucaProt represents an excellent tool for investigating protein-protein interactions between RNA-dependent RNA polymerase (RdRp) viruses and human hosts. LucaProt is a pLM specifically designed for RNA viral protein analysis. By integrating amino acid sequence data with structural information, the model employs a dual-channel architecture that processes sequence and structural features independently prior to concatenation for classification. This synergistic approach enables LucaProt to achieve enhanced detection accuracy for highly divergent RNA viruses, surpassing conventional methods in both sensitivity and specificity [24]. To further characterize viral proteins in our study, we leveraged LucaProt to generate fixed 256-dimensional feature vectors, thereby providing an alternative to ESM-2-based feature extraction methodologies.

AAindex provided quantitative descriptors of physicochemical attributes of 20 amino acids (e.g., hydrophobicity, charge) [25]. Following this methodology, we converted residues in human and viral proteins into numerical indices, enabling integration of biochemical properties into our framework.

### 2.4 Multimodal fusion module

To unify heterogeneous feature representations from sequence data and physicochemical properties, we used multimodal fusion integrated with a spatial alignment framework derived from Tumor Multi-Omics pre-trained Network (TMO-Net) [26]. This method harnesses the self-attention mechanism of the Transformer model to concurrently model intra-modality relationships within individual data types and inter-modality correlations across complementary biological features. This mechanism computes feature correlations and dynamically adjusts their weights through learned attention coefficients, enabling nuanced feature interaction analysis. Crucially, the architecture implements a hierarchical cross-modal attention system that explicitly bridges modality-specific representations through transformer layers. Following multi-layer Transformer processing, TMO-Net synthesizes a unified embedding space that coalesces specific and distinguishable patterns from all input modalities. This integrated representation encapsulates critical biological determinants essential for HV-PPI prediction, serving as input for downstream predictive tasks. The spatial alignment paradigm ensures isomorphic mapping of human and viral protein features—including sequence motifs and physicochemical descriptors—into a shared latent space, thereby enhancing the model capacity to discern evolutionary and biophysical correlations underlying HV-PPIs.

### 2.5 Downstream tasks

We conducted two distinct downstream tasks for prediction: binary classification and conditional sequence generation. The binary classification task was designed to determine the presence or absence of interactions in HV-PPI pairs, while the conditional generative task aimed to predict interacting protein sequences corresponding to a given protein.

For the binary classification task, we developed an output module incorporating a fully connected layer followed by a softmax activation function to estimate the interaction probability between human host and viral protein sequences. The resulting probability scores ranged from 0 to 1, with values closer to 1 indicating a higher likelihood of interaction.

For the conditional generation task, we employed a decoder-based architecture to predict missing protein sequences in HV-PPI pairs. Here we utilize the test data as the input to generate potential interacting human or viral sequences. Since model-generated sequences may not correspond to natural proteins, we retrieved 20,078 human reference protein sequences from National Center of Biotechnology Information (NCBI) (accession: GCF_000001405.40) and 17,451 reviewed viral protein sequences from UniProt Swiss-Pro (taxon ID 10239) to ensure biological relevance. Compared to metrics such as Euclidean distance, cosine similarity focuses more on the direction rather than the magnitude of embedded vectors [27,28]. This property exhibits greater stability in high-dimensional spaces and is especially well-suited for capturing semantic or functional similarities [29]. Through the application of cosine similarity, the “semantic consistency” between sequences generated by the model and real sequences can be assessed more effectively [30], thereby enhancing the biological plausibility and reliability of the generated proteins [31,32]. Therefore, we computed cosine similarity scores between the fused embeddings of inputs and the embeddings of these reference proteins. The five most similar reference sequences were selected as the final output to prevent the generation of artificial proteins.

### 2.6 Training phase

**Fig. 2:**
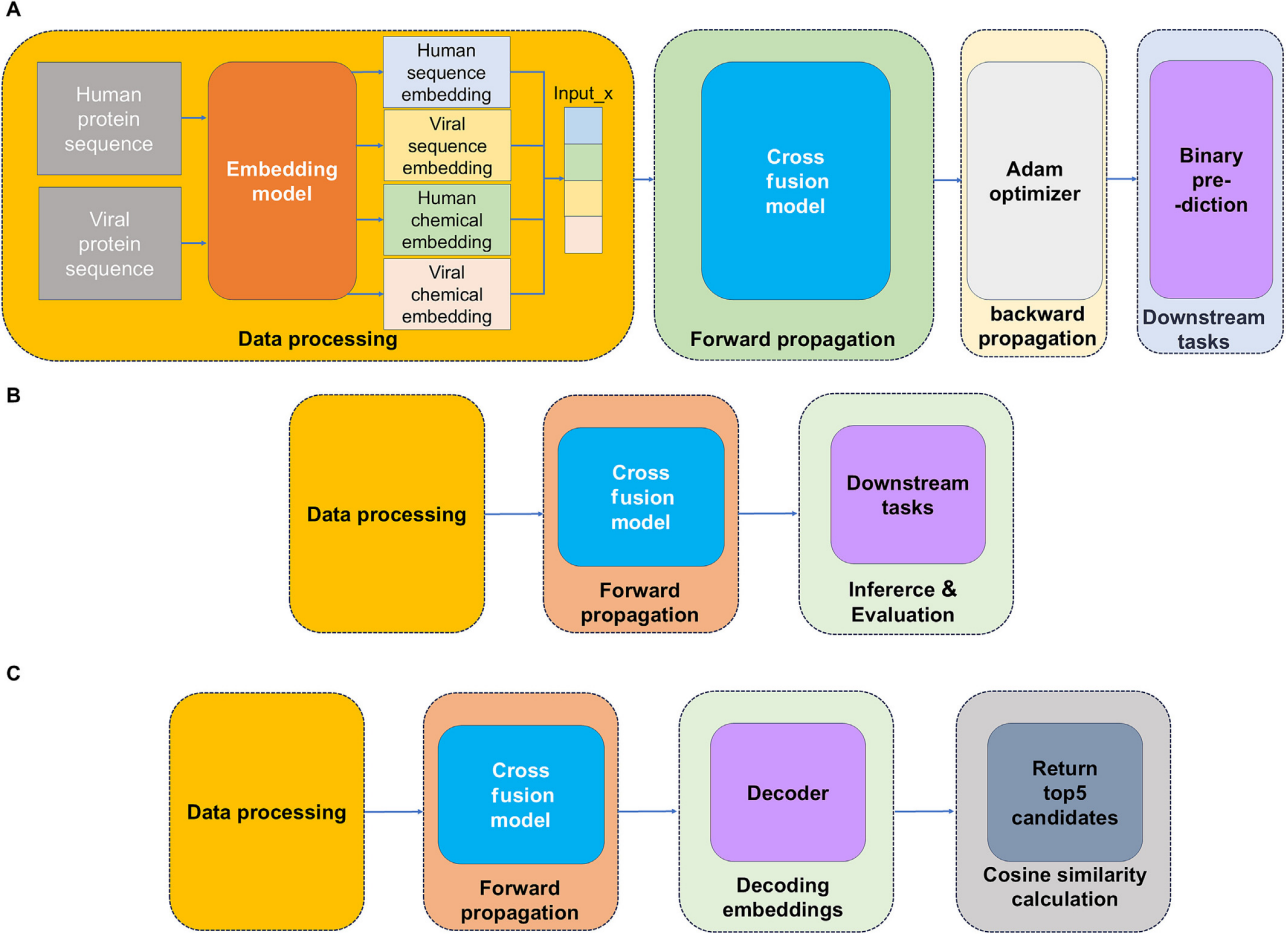
Schematic overview of training and inference workflows. A) Training phase: Positive and negative sample pairs are processed through the embedding module to generate four modality-specific embedding representations, human/virus sequence embedding is encoded by ESM-2 to represent amino acid sequence features, human/virus chemical embedding is extracted from AAindex profiles to represent protein biochemical properties. The cross-fusion module computes loss via contrastive learning and executes forward propagation. Model parameters are iteratively updated using the Adam optimizer, with final weights preserved for inference. B) Binary task inference. Pre-trained weights are loaded to compute task-specific losses, enabling downstream classification prediction. C) Conditional generative inference. A sequence decoder module translates fused modality embeddings into human protein sequences, with outputs ranked to return the top five highest-confidence matches.

All experiments were conducted on an NVIDIA A100 GPU (40 gigabyte) using Python 3.9.13 and PyTorch 1.13.1. Hyperparameters were initially selected based on those used in the original models and further optimized through empirical exploration of different combinations to identify the best-performing settings. For the binary classification task, models were trained for a maximum of 30 epochs with 3-fold cross-validation, employing a batch size of 16. The workflow (Fig. 2A) initiates by inputting human and viral protein sequences into an embedding module to extract semantic features, while physicochemical properties are concurrently derived by the AAIndex module. These distinct feature sets—semantic, structural, and biochemical—are integrated through a cross-fusion mechanism to generate unified protein representations. Training optimization utilized the Adam optimizer with a fixed learning rate of 0.0001, a loss weighting factor of 0.01, and no weight decay, aiming to minimize classification loss (Fig. 2B). Model performance was evaluated on the test set using the checkpoint achieving optimal validation results. For the conditional generative task, weights pretrained on the binary classification objective were transferred (Fig. 2C).

### 2.7 Ablation studies on model components

We hypothesized that the multimodal fusion module constitutes the primary contributor to DeepHVI performance enhancement. To test this hypothesis, we conducted controlled ablation studies by removing the multimodal fusion module from the original architecture. In this modified configuration, we implemented a fully connected layer immediately following the feature extraction module to perform binary classification, maintaining identical experimental conditions to those used during pre-training. To ensure comparability, we preserved the original hardware configuration and training epochs throughout both the ablation experiments and the subsequent inference phase.

### 2.8 Benchmark

We benchmarked DeepHVI against previous machine learning and deep learning approaches, including LucaOne [33], D-Script [34], xCAPT5 [35], and Topsy-Turvy [36] (Table 1), using identical test set evaluations. These approaches integrate both CNNs (convolutional neural networks), powerful deep learning algorithms commonly employed for analyzing visual data and utilized here to identify patterns within protein sequences, and GNNs (graph neural networks), a specialized class of neural networks adept at processing graph-structured data and particularly effective in capturing relationships between entities, such as proteins, represented as graphs. The comparative performance analysis was conducted using established binary classification metrics, providing a standardized framework for evaluating all models.

**Table 1:** Summary table of benchmarks.

Model Name	Approach	Primary application	Key features
LucaOne	Generalized biological foundation model	Integrates nucleic acid and protein sequence data	Unified deep learning framework for DNA and protein sequences
D-Script	Sequence-based, structure-aware prediction	PPI prediction	Uses CNNs and GNNs for genome-scale data processing
xCAPT5	Deep multi-kernel convolutional neural network	PPI prediction	Captures local and global protein sequence features
Topsy-Turvy	Graph convolutional networks combined with sequence data	PPI prediction	Incorporates global interaction context into PPI predictions

Abbreviations: CNN, convolutional neural network; GNN, graph neural network; PPI, protein-protein interaction; DNA, deoxyribonucleic acid.

## 3 Results

### 3.1 Feature extraction

**Fig. 3:**
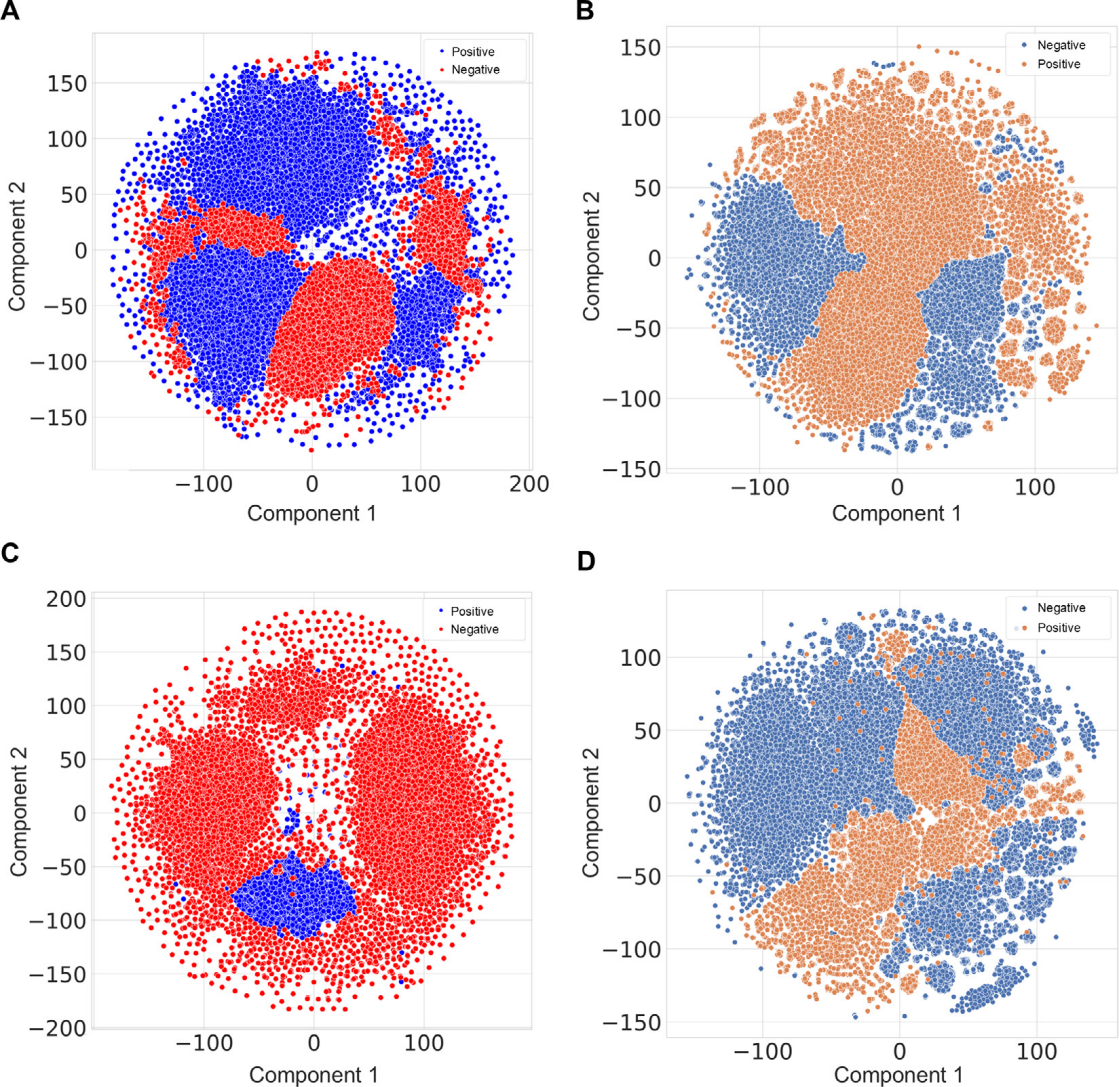
T-sne visualization of feature space distributions. A) Pre-fusion t-sne mapping of raw features in the ESM2-ESM2-AAindex configuration. B) Post-fusion t-sne mapping after cross-modal feature integration within the same framework. C–D) Analogous pre-fusion (C) and post-fusion (D) visualizations for the ESM2-Lucaprot-AAindex configuration.

To visualize embedding distributions and fused vector characteristics, t-distributed stochastic neighbor embedding (t-SNE) was used to project high-dimensional feature spaces onto a two-dimensional plane, enabling comparative analysis of data structure and cluster separation (Fig. 3). We evaluated two framework configurations for viral protein feature extraction: (1) both human and viral protein sequences were embedded by ESM-2 (ESM2-ESM2) (Fig. 3A, 3B); (2) and human proteins were embedded by ESM-2, while viral proteins were embedded by LucaProt (ESM2-LucaProt) (Fig. 3C, 3D). Physicochemical property features (AAindex) remained consistent across both configurations. Multimodal fusion substantially improved class separability, as fused vectors (Fig. 3B, 3D) showed clearer distinctions between positive and negative samples compared to direct feature stacking (Fig. 3A, 3C).

To quantify these improvements, silhouette scores were calculated for pre-fusion and post-fusion embeddings. The ESM2-ESM2-AAindex fused vectors achieved a mean silhouette score of 0.5390, a marked increase from the pre-fusion score of 0.4839, indicating enhanced intra-cluster cohesion and inter-cluster separation. This aligns with visual observations of reduced overlap between positive (host-viral interacting pairs) and negative (non-interacting) samples. In contrast, the ESM2-LucaProt-AAindex configuration showed more limited enhancement, with scores improving from 0.3746 to 0.4166, suggesting the critical influence of viral protein embedding model selection on fusion efficacy.

### 3.2 Binary classification prediction of interactions

The efficacy of the binary classification task for predicting interactions between HV-PPI pairs was assessed using standard performance metrics: accuracy, precision, recall, and F1-score (Table 2).

**Table 2:** Evaluation of binary classification prediction.

Model	Accuracy	Precision	Recall	F1	AUC
DeepHVI (ESM2-ESM2)	0.8107 ± 0.0616	0.8079 ± 0.2174	0.7636 ± 0.0982	0.7698 ± 0.1544	0.88
DeepHVI (ESM2-LucaProt)	0.8058 ± 0.0573	0.8303 ± 0.2110	0.7575 ± 0.1011	0.7703 ± 0.1550	0.87
DeepHVI (ESM2-ESM2, without fusion)	0.7303	0.7465	0.8952	0.8141	0.84
DeepHVI (ESM2-LucaProt, without fusion)	0.6619	0.6614	0.9993	0.7959	0.75
LucaOne	0.6900	0.7000	0.9700	0.8100	0.80
xCAPT5	0.6898	0.7022	0.9656	0.8130	0.80
D-Script	0.2983	0.7444	0.0144	0.0283	0.55
Topsy-Turvy	0.3505	0.7539	0.1235	0.2123	0.60

Abbreviation: AUC, area under curve.

The DeepHVI framework demonstrated superior discriminative performance, achieving high accuracy and precision. Its precision highlights the model capacity to minimize false positives—a critical advantage in biological studies, as erroneous predictions could necessitate costly experimental follow-ups. Moreover, the close alignment of its F1-score, accuracy, recall, and precision suggests a well-balanced performance, indicating that the model effectively reconciles sensitivity and specificity without disproportionately prioritizing either metric. This alignment is particularly helpful in HV-PPI prediction, where class imbalance or dataset bias frequently compromises model robustness.

To benchmark DeepHVI against existing approaches, we evaluated LucaOne, D-Script, xCAPT, and Topsy-Turvy under identical experimental conditions. In contrast to our framework, these benchmark methods exhibited marked variability across performance metrics. For instance, while LucaOne and xCAPT achieved high recall scores, their precision and accuracy lagged significantly, reflecting a propensity for generating false positives or misclassifying negative instances. Such disparities suggest limitations in the generalizability of the benchmark models to diverse HV-PPI interaction patterns or their ability to capture nuanced biological features essential for accurate classification. Collectively, the integration of all four metrics demonstrates that DeepHVI achieves superior overall performance in predicting human-viral protein interactions.

Model performance evaluation was evaluated using four metrics. (1) Accuracy: Proportion of correctly predicted interacting/non-interacting pairs among all samples, reflecting overall discriminative capability; (2) Precision: Ratio of true interacting pairs to all predicted positives, quantifying the model’s ability to minimize false positives (mislabeling non-interacting pairs as interacting); (3) Recall: Proportion of true interacting pairs successfully identified, indicating sensitivity to avoid missing true interactions (false negatives); (4) F1-score: Harmonic mean of precision and recall, balancing robustness in class-imbalanced data. The standard deviation of the metrics is also shown for the DeepHVI models. However, for other models in the benchmarking, there is no standard deviation since cross-validation was not performed.

### 3.3 Ablation experiments of binary prediction

Ablation experiments on the binary prediction task demonstrate the critical importance of the multimodal fusion module in effectively capturing protein features of HV-PPIs. To evaluate the impact of the fusion module, this component was systematically removed from the model architecture, and the resulting performance was compared to the original framework. The removal precipitated a marked decline in accuracy and precision (Table 2), with accuracy decreasing by 9.92 % and 18.86 %, and precision dropping by 7.60 % and 20.34 %, respectively. These reductions underscore the role of the fusion module in reducing misclassifications. Additionally, observed variations in the performance metrics suggested that the module mitigates class imbalance—a common challenge in binary classification tasks involving heterogeneous biological data.

### 3.4 Conditional generative prediction of interacting sequences

**Fig. 4:**
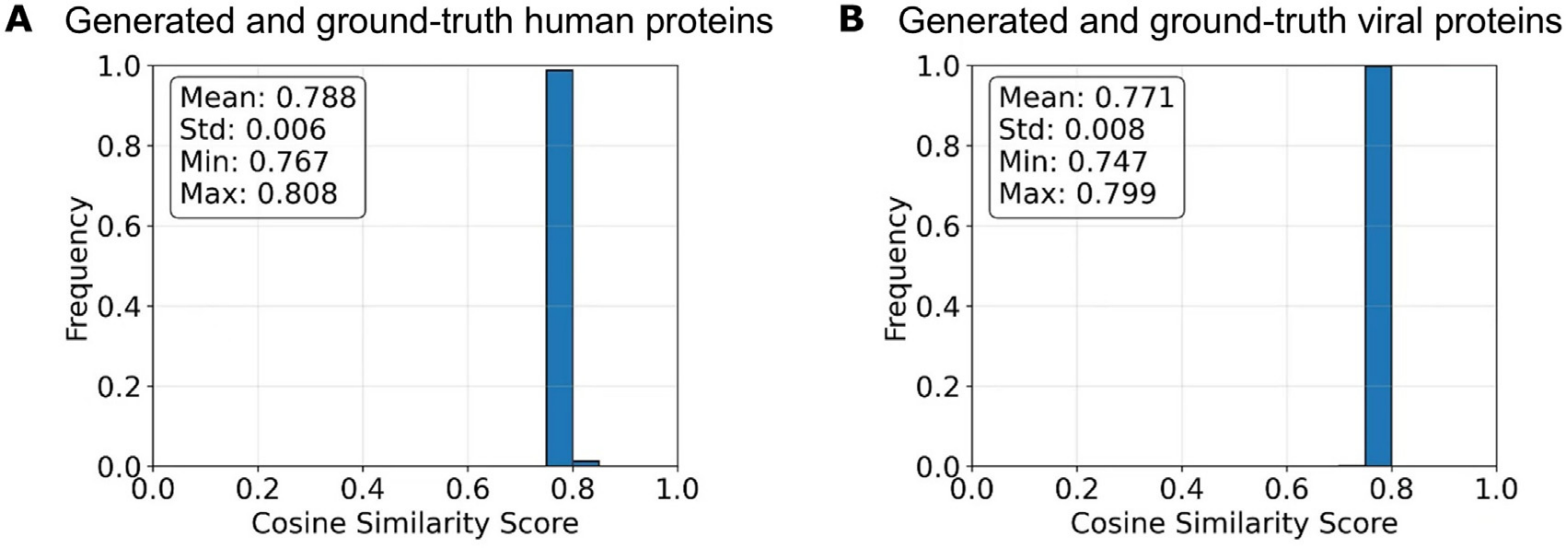
Cosine similarity analysis of generated human protein sequences and viral protein sequences. A) Cosine similarity between generated and ground-truth human proteins, illustrating the distribution of cosine similarity values between human protein sequences. B) Cosine similarity between generated and ground-truth viral proteins, depicting the distribution for viral protein sequences, which exhibits slightly lower and more variable similarity scores. In both cases, the distributions are sharply peaked around 0.8, indicating a generally strong semantic alignment across samples. Human and viral protein sequences from the test set were analyzed using DeepHVI to generate a density distribution of similarity scores between reconstructed sequences and human interactors. Abbreviations: Std, standard deviation; Min, minimum; Max, maximum.

While binary classification methods can identify potential protein-protein interactions by screening novel viral proteins against all human proteins, this approach incurs substantial computational costs. To address this limitation, we additionally developed a conditional generative task capable of generating protein sequences interacting with a given protein. To assess its predictive performance, we extracted proteins from the test set and generated putative interacting protein sequences from the model. We then computed the cosine similarity between these generated sequences and the experimentally validated interacting human or viral sequences in the test set. The model attained a high average similarity score of 0.788 ± 0.006 in the task of generating human sequences (Fig. 4A) and an average similarity score of 0.771 ± 0.008 in the task of generating viral sequences (Fig. 4B), indicating strong alignment with ground-truth interactions. This demonstrates the utility of the conditional generative approach in predicting biologically relevant interactions with improved efficiency, offering a resource-effective alternative to exhaustive screening methods.

### 3.5 Case study of conditional generative prediction

To demonstrate the effectiveness of the conditional generative task, we analyzed 28 SARS-CoV-2 protein sequences not included in our training dataset (Table S1). Generated human protein sequences were clustered using the STRING database visualized by Cytoscape [37,38], revealing five host proteins with previously documented interactions with SARS-CoV-2 components. Large-scale omics screening indicates that SARS-CoV-2 interacts with IL17RA. We identified that IL17 binds with IL17RA to form a heterodimeric complex, leading to the induction of expression of inflammatory chemokines and cytokines [39]. Mass spectrometry analyses further identified an interaction between the viral membrane (M) protein and PSMA4, a proteasome subunit, suggesting a mechanism for immune evasion through proteasomal interference [40]. RNA-protein interactomics studies demonstrate that nonstructural protein 1 (Nsp1) binds RPA1, a key player in DNA repair, implying suppression of the host’s DNA damage response [41]. Computational analyses combining network pharmacology and pathway enrichment predicted potential interference of the Spike protein with the Wnt signaling pathway (including WNT7A) [42], findings that align with our machine learning-driven predictions.

Although our findings are consistent with previously published literature, over half of the identified interactions lack direct experimental evidence confirming their association with SARS-CoV-2. Nevertheless, through an in-depth review of existing literature, we found that these proteins are indeed involved in multiple pathways associated with viral host invasion. For instance, we also identified P2X4, a member of the P2X receptor family, as a critical regulator of calcium ion channels. Viruses may disrupt the calcium signaling pathway, thereby altering the intracellular environment to facilitate optimal viral replication [43]. These findings collectively highlight mechanisms by which SARS-CoV-2 proteins may subvert host pathways through direct molecular interactions.

**Fig. 5:**
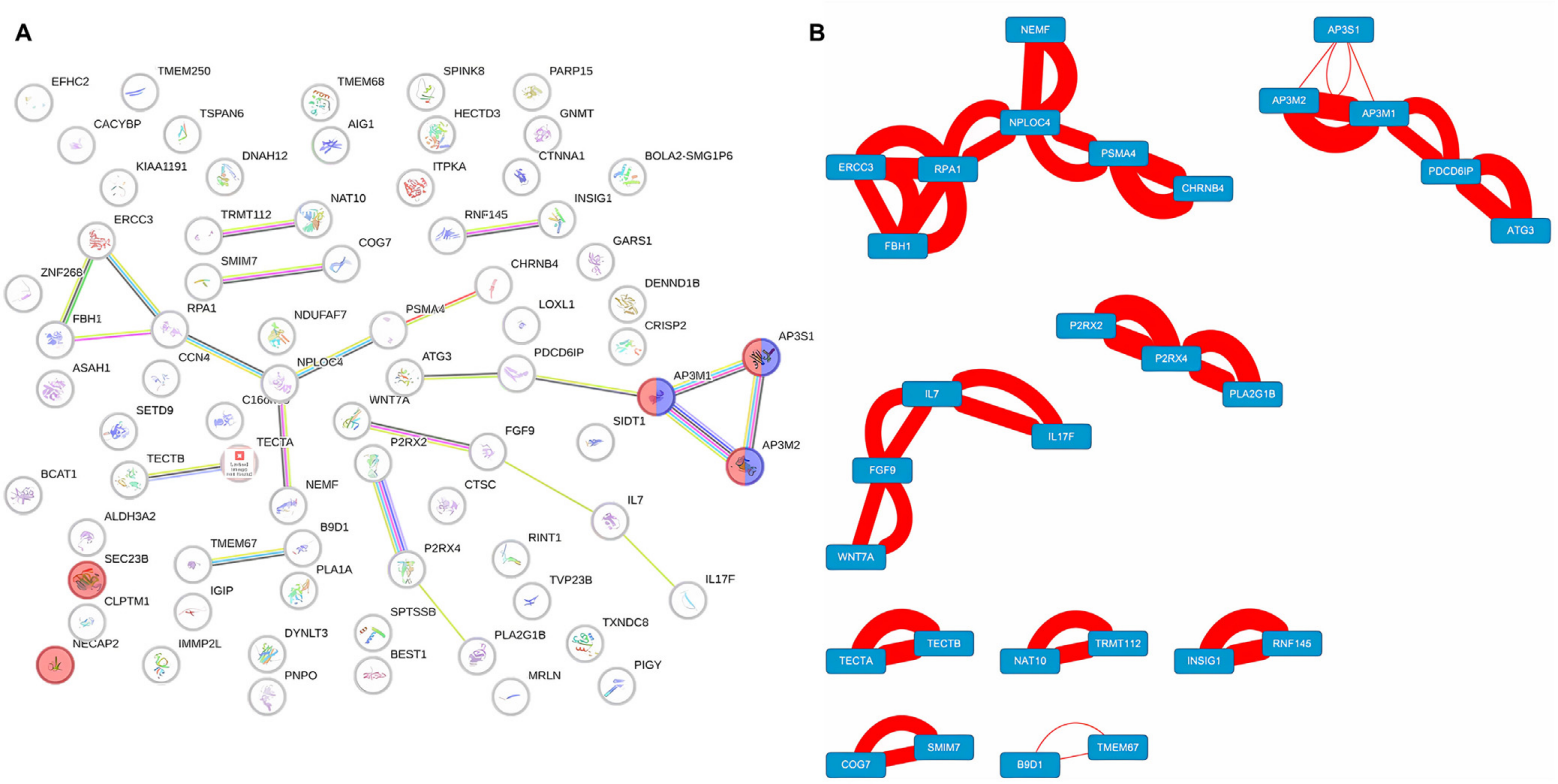
STRING-based clustering analysis of model-generated human proteins. A) Predicted candidate proteins were functionally analyzed using STRING to identify known interactions documented in the literature. B) The interaction network was visualized in Cytoscape, where each node represents a protein and edges denote their interactions. Edge thickness reflects confidence level, with thicker edges indicating stronger or more reliable predicted associations.

We further conducted a network analysis of the identified interacting proteins (Fig. 5). In the protein network, two major functional modules were mainly observed: cell complex-related proteins and membrane coating-related proteins. Among them, the cell complex-related proteins mainly included the AP-3 adaptor complex (AP3S1, AP3M1, AP3M2), while the membrane coating-related proteins included SEC23B and NECAP2. Previous studies have shown that the subunit AP3B1 of the AP-3 complex interacts with the E protein (envelope protein) of the novel coronavirus [39], suggesting that the AP-3 complex may be involved in the endocytosis of viral particles, especially playing a significant role in specific cell types such as immune cells. NECAP2 may promote the entry of the novel coronavirus into host cells through the ACE2 receptor by assisting in clathrin-mediated endocytosis [44,45]. Additionally, SEC23B, as a key component of the COPII complex, may facilitate the protein transport of the novel coronavirus within host cells and the assembly of viral particles by participating in the protein transport pathway from the endoplasmic reticulum to the Golgi apparatus [46,47].

## 4 Discussion

The DeepHVI framework advances HV-PPI prediction by integrating state-of-the-art deep learning architectures with multimodal learning strategies. PLMs enable the capture of intricate biological signatures within protein sequences, while physicochemical properties provide complementary insights [48]. A multimodal fusion architecture synthesizes these heterogeneous data types, generating a multidimensional representation of protein pairs that significantly enhances prediction reliability [49]. Collectively, these components form a robust computational framework for accurately predicting potential interactions, offering novel insights into viral pathogenesis.

To address distinct use cases in HV-PPI prediction, we proposed two complementary computational tasks. The first uses binary classification to determine interaction likelihood between a given pair of host and viral proteins. The second, a conditional sequence generation task, predicts candidate human protein interactors for a specified viral protein, overcoming a critical limitation of traditional PPI studies. Conventional approaches usually focused solely on pairwise interaction prediction, necessitating exhaustive pairwise analysis of all human proteins to identify potential viral targets [50]. This imposes prohibitive computational demands, particularly for novel pathogens. To resolve this, our framework introduced a conditional generative task that directly infers plausible human interactors. To ensure biological validity and biosafety, generated candidates are filtered through a similarity search strategy, returning natural proteins as output and preventing the generation of artificial proteins. This dual-task design not only enhances computational efficiency but also enables rapid deployment in emerging epidemic scenarios.

While benchmark datasets designed for PPI conditional generation tasks are currently unavailable, empirical validation in real-world applications underscores the utility of our approach. In a representative case study, our method identified multiple candidate interactions that align with experimentally confirmed PPIs reported in prior literature [39,41,42,51,52]. While some of these results align with previous findings, a substantial proportion of the interactions predicted by DeepHVI extend beyond the capabilities of traditional methods. This demonstrates the superior ability of machine learning to detect complex and non-linear patterns in PPIs, and underscores the potential of DeepHVI to uncover novel interactions that have yet to be experimentally validated. These findings emphasize the capacity of our method for rapid adaptation to emerging pathogens, providing a streamlined framework to accelerate the characterization of unknown viral protein functions during outbreak scenarios.

DeepHVI primarily leverages deep learning models to PPIs and, in principle, demonstrates superior capabilities in discerning functional differences compared to traditional homology-based methods. However, accurately distinguishing true interactions from false positives among highly homologous proteins remains a significant challenge. The generalization capacity of DeepHVI fundamentally depends on the diversity of its training data and the effectiveness of model regularization techniques. Due to the limited size of our current dataset, we have not yet implemented specific strategies to mitigate homology bias (e.g., stratified sampling to reduce sequence similarity within the training set). As a result, DeepHVI may exhibit an elevated rate of false positives in scenarios involving high homology. Moving forward, we aim to incorporate more diverse PPI datasets and validate the model’s performance on highly homologous proteins through additional experimental evidence.

The DeepHVI model also holds promise for facilitating vaccine design and antiviral drug discovery by adapting to various types of input data. Given a sufficient number of paired datasets, the model can learn task-specific weights during training and subsequently apply these weights to sequence generation tasks as well as binding prediction. Intrinsically disordered regions (IDRs) are prevalent in viral proteins, and their dynamic conformations play critical roles in protein function. However, modeling IDRs using pLMs remains challenging due to issues such as structural database bias, inadequate representation of conformational dynamics, and polymorphism. In the future, there is great potential to construct IDR-specific training sets by integrating experimental data (e.g., hydrogen–deuterium exchange mass spectrometry) with enhanced sampling algorithms (e.g., MetaDynamics). Additionally, the development of generative pLMs capable of producing conformational probability distributions (e.g., diffusion models) holds promise for providing improved solutions for modeling IDRs.

While demonstrating substantial innovation, DeepHVI has limitations that merit further investigation. First, its dependence on existing experimental datasets may propagate biases stemming from inherent gaps in viral and host protein diversity coverage. Expanding taxonomic representation and integrating continuously updated experimental datasets could mitigate these biases and enhance generalizability. Second, while the framework successfully combined sequence-based and physicochemical features, it omits explicit integration of structural or dynamic protein properties—critical determinants of interaction mechanisms. Future iterations could address this gap by incorporating structural modeling techniques (e.g., AlphaFold-predicted structures) or experimentally resolved structural data, which may substantially improve predictive accuracy.

Ethical considerations remain central to the responsible use of AI for pandemic preparedness. Ensuring transparency and validating model predictions are essential steps before advancing to clinical or public health applications. This study adheres to these principles and guidelines to ensure the development of a safe and ethical approach for use in sensitive domains. In accordance with biosafety considerations, only naturally occurring proteins are generated, and no artificially designed proteins are produced at any stage.

## 5 Conclusion

In summary, DeepHVI establishes a computational framework for predicting HV-PPIs, addressing challenges in virology research. By enabling rapid identification of interaction candidates, this method advances mechanistic studies of viral pathogenesis while providing empirically grounded insights for systematically characterizing host-pathogen interaction networks. Its capacity for fundamental discovery and translational applications positions DeepHVI as a critical resource for virus and pandemic studies.

## Figures and Tables

**Supplementary Fig. 1:**